# SARS-CoV-2 Infection, Hospitalization, and Associated Factors Among People Living With HIV in Southeastern China From December 2022 to February 2023: Cross-Sectional Survey

**DOI:** 10.2196/51449

**Published:** 2024-04-17

**Authors:** Wei Cheng, Yun Xu, Haibo Jiang, Jun Li, Zhigang Hou, Haibin Meng, Wei Wang, Chengliang Chai, Jianmin Jiang

**Affiliations:** 1 Zhejiang Provincial Center for Disease Control and Prevention Hangzhou China; 2 Ningbo Center for Disease Control and Prevention Ningbo China; 3 Wenzhou Center for Disease Control and Prevention Wenzhou China; 4 Jiaxing Center for Disease Control and Prevention Jiaxing China; 5 Shaoxing Center for Disease Control and Prevention Shaoxing China; 6 Quzhou Center for Disease Control and Prevention Quzhou China

**Keywords:** associated factors, COVID-19, hospitalization, infection, people living with HIV, SARS-CoV-2 Omicron variant

## Abstract

**Background:**

Limited studies have explored the impact of the Omicron variant on SARS-CoV-2 infection, hospitalization, and associated factors among people living with HIV, particularly in China. The adjustment of preventive policies since December 2022 in China presents an opportunity to evaluate the real-world factors influencing SARS-CoV-2 infection and related hospitalization among people living with HIV.

**Objective:**

This study aimed to investigate SARS-CoV-2 infection, hospitalization rates, and associated factors among people living with HIV following the adjustment of preventive policies from December 2022 to February 2023 in southeastern China.

**Methods:**

A cross-sectional telephone or web-based survey was conducted among people living with HIV in 5 cities in southeastern China from December 2022 to February 2023. Demographic information, SARS-CoV-2 infection and related hospitalization, and HIV-specific characteristics were collected from existing databases and special investigations. Multivariate logistic regression analyses were conducted to determine the associated factors for infection and hospitalization rates of SARS-CoV-2. Additionally, subgroup analyses were conducted for the association between vaccination and infection across different vaccination statuses and time since the last vaccination.

**Results:**

Among people living with HIV with a COVID-19 testing history, the SARS-CoV-2 infection rate was 67.13% (95% CI 65.81%-68.13%), whereas the hospitalization rate was 0.71% (95% CI 0.46%-0.97%). Factors such as age, latest CD4 cell count, latest HIV viral load, and transmission route were found to be associated with SARS-CoV-2 infection, while age, cancer, latest CD4 cell count, and latest HIV viral load were associated with SARS-CoV-2 hospitalization. In terms of SARS-CoV-2 vaccination, compared to unvaccinated people living with HIV, there was a lower infection rate among those who had been vaccinated for <3 months in the booster vaccination group (adjusted odds ratio [aOR] 0.72, 95% CI 0.53-0.98; *P*=.04); and there was also a lower risk of hospitalization among people living with HIV who had received vaccination in the past 6-12 months (aOR 0.33, 95% CI 0.14-0.81; *P*=.02) and more than 12 months ago (aOR 0.22, 95% CI 0.07-0.72; *P*=.01).

**Conclusions:**

After the ease of prevention and control measures in China, we observed a high SARS-CoV-2 infection rate but a low hospitalization rate. General risk factors, such as higher age and vaccination status, and HIV-related parameters, such as the latest CD4 cell count and HIV viral load, were associated with SARS-CoV-2 infection and hospitalization. A booster vaccination campaign for booster doses should be considered among people living with HIV in confronting possible COVID-19 epidemic emergencies in the near future.

## Introduction

HIV/AIDS has remained a significant global public health threat despite being discovered over 40 years ago. Currently, there are 38 million people living with HIV worldwide, and in 2021 alone, 650,000 individuals died from HIV-related illnesses [1]. The situation regarding global HIV epidemic control has become more severe due to the emergence of the COVID-19 pandemic caused by SARS-CoV-2 [2,3]. It can be understood that the weakening of the immune system of patients with HIV/AIDS increases the possibility of severe outcomes from SARS-CoV-2 coinfection. However, there is limited data on the susceptibility and vaccine effectiveness of people living with HIV to SARS-CoV-2 infection and severe outcomes. As there is no cure for HIV and there is a chance of SARS-CoV-2 infection, coinfection continues to pose a problem [3-5].

Previous studies have suggested that older age and comorbidities such as hypertension, diabetes, and cardiovascular disease are risk factors for developing severe COVID-19 and are associated with high mortality rates [6]. However, the interaction of people living with HIV–specific factors, such as CD4 cell count, HIV viral load, and antiretroviral treatment (ART), with COVID-19 remains inclusive [3,6-8]. Moreover, most of these studies were conducted before the vaccination or during the epidemics of the Alpha and Delta variants. Limited studies have explored the impact of the Omicron variant, which exhibits increased transmissibility and immune escape abilities, on people living with HIV [9]. Furthermore, because of the strict prevention and control policies for COVID-19 in China, few studies concerning the SARS-CoV-2 infection and hospitalization among people living with HIV have been reported in the country.

Due to a unified, tightly coordinated COVID-19 vaccination campaign during the dynamic COVID-19 Zero period, partial and full vaccination coverage in China reached 91.5% and 89.3%, respectively [10,11]. In the context of this high vaccination coverage and the relatively weak virulence of the SARS-CoV-2 Omicron variant, the Chinese government relaxed COVID-19 prevention and control measures in December 2022. Notably, several studies reported a significant surge in cases between December 2022 and January 2023, with reported infection rates ranging from 40.9% to 92.3% in the general population [11-15]. However, there are currently no reports regarding SARS-CoV-2 infection and hospitalization among people living with HIV during this period. Moreover, although a few studies demonstrate the short-duration protection of the COVID-19 vaccine against infection in the general population [11,12,16,17], there was no study on the impact of the COVID-19 vaccine on infection and hospitalization of SARS-CoV-2 among people living with HIV in China. The substantial epidemic presents an opportunity to evaluate the real-world factors influencing the infection and hospitalization of SARS-CoV-2 among people living with HIV. Therefore, this study investigated the infection and hospitalization of SARS-CoV-2 and identified associated factors among people living with HIV from December 2022 to January 2023.

## Methods

### Study Population

Zhejiang Province is located in southeastern China and is known for its thriving economy and high population density. This province is home to over 65 million local residents spread across 11 metropolitan areas.

**Figure 1:**
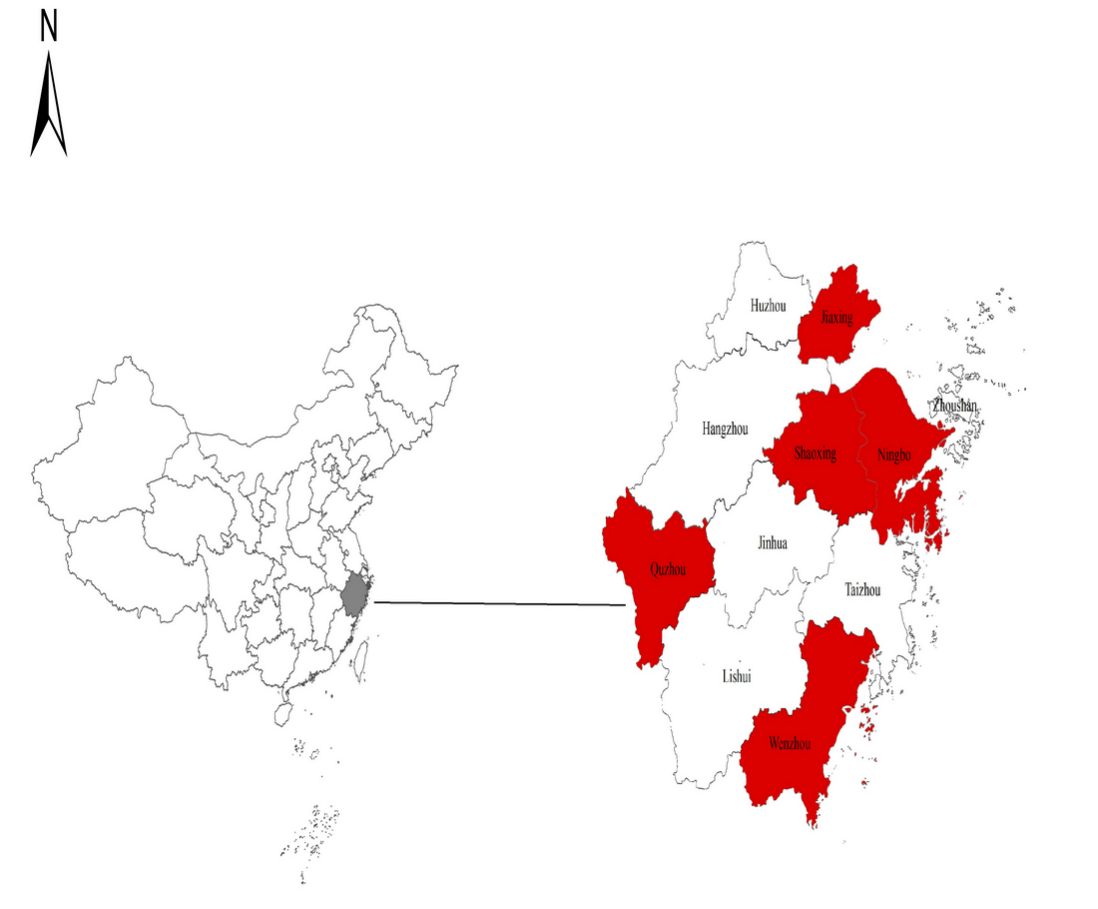
Geographical location of 5 study sites in eastern China.

By the end of 2022, the total number of people living with HIV living in Zhejiang Province was 39,744. For our study, we selected 5 metropolitan areas in Zhejiang province as our study sites: Ningbo in the east, Quzhou in the west, Jiaxing in the north, Wenzhou in the south, and Shaoxing in the middle of the province (Figure 1). The total number of people living with HIV in the 5 cities was unbalanced, with Ningbo the highest (6096) and Quzhou the smallest (1346). As a result, the 5 selected cities accounted for 46% of all living cases of people living with HIV in the entire province.

Our target sample size was 10,000, which was estimated by experts. Center for Disease Control and Prevention (CDC) staff from the 5 cities downloaded the database from the HIV/AIDS case reporting information system, established by the Chinese CDC. Considering the work foundation of each city, we randomly selected 50% (5933/11,865) of people living with HIV from Wenzhou and Ningbo and 80% (5102/6378) from Quzhou, Jiaxing, and Shaoxing as potential participants (total number=11,035) to be investigated. The inclusion criteria for people living with HIV in the final analysis were as follows: (1) be aged ≥15 years; (2) currently live in the abovementioned 5 cities; and (3) agree to participate in the survey. Exclusion criteria mainly included people living with HIV who were not currently taking ART or who did not have results of CD4 cell count and HIV viral load.

### Data Collection

To collect information regarding SARS-CoV-2 infection, admission status, and related symptoms, a questionnaire was designed by the Zhejiang Provincial CDC. The investigation was conducted by trained county CDC staff between January 16 and February 10, 2023. Uniform training was organized by the Zhejiang CDC and provided to investigators to ensure the quality of the investigation. The method of investigation was determined by the investigators. Ningbo staff conducted a web-based survey using Questionnaire Star (one of the most widely used commercial web-based survey platforms in China). Staff from 4 other cities conducted an investigation by telephone. During the investigation, we first inquired whether the participants had experienced any COVID-19–related symptoms since December 7, 2022. Subsequently, we asked if they had tested positive for the SARS-CoV-2 infection. If they responded with a “yes” or “no,” we further asked for the method of confirmation, specifically whether it was through an antigen test or nucleic acid test. For those who self-reported having SARS-CoV-2 infection, we asked for the confirmation date and whether they had been hospitalized after the infection. We further investigated the reasons for hospitalization for individuals admitted to the hospital.

Demographic information such as age, sex, and marital status was collected from the HIV/AIDS case reporting information system. Information related to ART, such as the initiation date, the latest CD4 cell count, viral load, and treatment regime, was obtained from the national HIV treatment subdatabase of Zhejiang province. Regarding the ART regime, we focused on the national free drugs that are widely used among people living with HIV in China, such as tenofovir (TDF), azidothymidine (AZT), lamivudine (3TC), efavirenz (EFV), and lopinavir and ritonavir (LPV/r).

COVID-19 vaccine–related data, including vaccine type, vaccine dose, and last vaccination date, were obtained from the vaccination information system. In terms of vaccine brand and manufacturer, an inactivated vaccine was produced by Sinopharm CNBG Beijing, Sinopharm CNBG Wuhan and the Beijing-based Sinovac Biotech; a recombinant protein (Chinese hamster ovary [CHO] cell) vaccine was produced by Anhui Zhifei Longcom Biopharmaceutical Institute of Microbiology; and an adenovirus vector vaccine was used, referred to as Cansino Ad5-nCoV-S COVID-19 vaccine.

### Definition

Due to the absence of COVID-19 test results for all participants at the time of investigation, we categorized the study participants into groups A, B, and C. Group A consisted of individuals with no history of COVID-19 testing but reported COVID-19–related symptoms. Among these participants, those who presented with symptoms such as fever or cough were classified as having SARS-CoV-2 infection, while those who did not exhibit such symptoms were categorized as noninfected. Group B comprised individuals who had undergone COVID-19 testing between December 7, 2022, and the investigation date. Infection status was determined based on the positive or negative results of nucleic acid or antigen tests. Group C encompassed all study participants, including groups A and B.

Hospitalizations that were unlikely to be attributed to COVID-19, such as cases involving non–COVID-19 infections or long-term hospitalizations, were excluded from the study.

According to the Technical Vaccination Recommendations for COVID-19 Vaccines in China and previous studies [16-18], vaccination status was categorized into 4 levels: (1) unvaccinated, which indicates no history of COVID-19 vaccination before the last SARS-CoV-2 exposure date; (2) partially vaccinated, which includes people who have received 1 dose of inactivated vaccine, received 2 doses of inactivated vaccine but received the second dose within 14 days before the last SARS-CoV-2 exposure date, received 2 doses of recombinant protein vaccine (3 doses are recommended for full primary vaccination), and received 1 dose of adenovirus vector vaccine, or 3 doses of recombinant protein vaccine but with the last dose within 14 days before the last SARS-CoV-2 exposure date; (3) full primary vaccination, which includes people who received either 2 doses of inactivated vaccine, 1 dose of adenovirus vector vaccine, or 3 doses of recombinant protein (CHO cell) vaccine with the last vaccination 14 days or more before the last SARS-CoV-2 exposure date and with no booster dose; 2 doses of inactivated vaccine with 1 booster dose of inactivated vaccine, adenovirus vector vaccine, or recombinant protein (CHO cell) vaccine within 7 days before the last SARS-CoV-2 exposure date; or 2 doses of adenovirus vector vaccine within 7 days before the last exposure date; (4) booster vaccinated, which includes people who received either 2 doses of Ad5-vector vaccine with the second dose 7 days or more before the last exposure date; or 2 doses of inactivated vaccine and 1 or 2 booster doses of inactivated vaccine, adenovirus vector vaccine, or recombinant protein vaccine 7 days or more before the last exposure date.

The time since the last vaccination was calculated as the interval between the date of the last vaccination dose and the date of the last SARS-CoV-2 exposure. The date of last SARS-CoV-2 exposure was defined as the date of the investigation if the participants had not self-reported SARS-CoV-2 infection; otherwise, it was the date of the positive result of self-tested SARS-CoV-2.

### Statistical Analysis

The means (SDs) or medians (IQRs) were obtained for continuous variables. Categorical variables were analyzed by calculating characteristic percentages. To analyze the associated factors for SARS-CoV-2 infection, logistic regression analysis was performed among those with a history of SARS-CoV-2 test (group B). To identify risk factors for hospitalization, a multivariable logistic regression analysis was conducted among participants who had undergone SARS-CoV-2 testing and received a positive result. Univariate factors with a significance level of *P*<.10 and factors previously identified to be associated with COVID-19 infection were included in the full multivariable regression models. Additionally, to make the results more robust, we conducted a sensitivity analysis for the association between SARS-CoV-2 infection rate and vaccination among groups A and C. Data cleaning and organization were performed using Microsoft Excel 2016 software (Microsoft Corporation). Statistical analysis was conducted using R statistical software (version 4.1.2, R Project for Statistical Computing). A significance level of *P*<.05 was considered statistically significant in the final model.

### Ethics Statement

The study was approved by the ethical review board of the Zhejiang Provincial CDC (IRB approval number: 2023-003-01). No risk was involved in participating in this study, and we protected the confidentiality of the participants. For those participants aged 18 years or younger, we sought parental consent and collected data from their parents.

## Results

### Characteristics of the Study Participants

In total, 9922 people living with HIV (group C) were included in the final analyses, with 3628 classified as group A and 6294 as group B. The response rate was 89.91% (9922/11,035) for the potential participants and 24.96% (9922/39,744) for all people living with HIV in the province. The median age of the participants was 46 years (IQR 22), of which 7996 (80.59%) were male. Among them, 6848 (69.02%) were registered residents of Zhejiang Province. The prevalence of chronic diseases among the participants was as follows: hypertension (930/9992, 9.37%), diabetes 395/9922, 3.98%), and cardiovascular diseases (103/9922, 1.04%). Heterosexual transmission accounted for a higher percentage (5825/9922, 58.71%) compared to homosexual transmission (4001/9922, 40.32%). The median latest CD4 count was 462 cells/μL (IQR 301), and the median ART duration was 5.38 years (IQR 4.65). Among the people living with HIV, 9274 (93.47%) had an HIV viral load below 50 copies/mL. Regarding vaccination status, 1530 (15.42%) were unvaccinated, 455 (4.59%) had received their last vaccination less than 3 months ago, 237 (2.39%) had received their last vaccination between 3 and 5 months ago, 4707 (47.44%) had received their last vaccination between 6 and 11 months ago, and 2993 (30.17%) had received their last vaccination more than 12 months ago (Table 1). Among the entire study population, the proportion of individuals with partial vaccination, full primary vaccination, and booster vaccination was 2.59%, 15.32%, and 66.67%, respectively (Table 1).

**Table 1:** Characteristics of the study participants among people living with HIV in southeastern China from December 2022 to February 2023.

Variable			Group A (n=3628)	Group B (n=6294)	Group C (n=9922)
Age (years), median (IQR)			49 (22)	44 (23)	46 (22)
**Age categories (years), n (%)**					
	15-29		362 (9.98)	955 (15.17)	1317 (13.27)
	30-39		720 (19.85)	1671 (26.55)	2391 (24.1)
	40-49		807 (22.24)	1254 (19.92)	2061 (20.77)
	50-59		923 (25.44)	1397 (22.2)	2320 (23.38)
	≥60		816 (22.49)	1017 (16.16)	1833 (18.47)
**Sex, n (%)**					
	Male		2813 (77.54)	5183 (82.35)	7996 (80.59)
	Female		815 (22.46)	1111 (17.65)	1926 (19.41)
**Education, n (%)**					
	Illiteracy		208 (5.73)	227 (3.61)	435 (4.38)
	Primary school		822 (22.66)	1065 (16.92)	1887 (19.02)
	Middle school		1462 (40.3)	2195 (34.87)	3657 (36.86)
	High school or technical secondary school		633 (17.45)	1356 (21.54)	1989 (20.05)
	College degree or above		503 (13.86)	1451 (23.05)	1954 (19.69)
**Marital status, n (%)**					
	Single or divorced		1701 (46.89)	3589 (57.02)	5290 (53.32)
	Married or cohabiting		1922 (52.98)	2695 (42.82)	4617 (46.53)
	Unknown		5 (0.14)	10 (0.16)	15 (0.15)
**Household registration, n (%)**					
	Zhejiang province		2494 (68.74)	4354 (69.18)	6848 (69.02)
	Other provinces		1134 (31.26)	1940 (30.82)	3074 (30.98)
**Hypertension, n (%)**					
	Yes		363 (10.01)	567 (9.01)	930 (9.37)
	No		3265 (89.99)	5727 (90.99)	8992 (90.63)
**Diabetes, n (%)**					
	Yes		133 (3.67)	262 (4.16)	395 (3.98)
	No		3495 (96.33)	6032 (95.84)	9527 (96.02)
**Chronic obstructive pneumonia, n (%)**					
	Yes		12 (0.33)	19 (0.3)	31 (0.31)
	No		3616 (99.67)	6275 (99.7)	9891 (99.69)
**Cardiovascular diseases, n (%)**					
	Yes		40 (1.1)	63 (1)	103 (1.04)
	No		3588 (98.9)	6231 (99)	9819 (98.96)
**Chronic kidney disease, n (%)**					
	Yes		20 (0.55)	27 (0.43)	47 (0.47)
	No		3608 (99.45)	6267 (99.57)	9875 (99.53)
**Cancer, n (%)**					
	Yes		18 (0.5)	38 (0.6)	56 (0.56)
	No		3610 (99.5)	6256 (99.4)	9866 (99.44)
**Cerebrovascular disease, n (%)**					
	Yes		15 (0.41)	27 (0.43)	42 (0.42)
	No		3613 (99.59)	6267 (99.57)	9880 (99.58)
**Transmission route, n (%)**					
		Heterosexual	2322 (64)	3503 (55.66)	5825 (58.71)
		Homosexual	1268 (34.95)	2733 (43.42)	4001 (40.32)
		Other or unknown	38 (1.05)	58 (0.92)	96 (0.97)
Latest CD4 count (cells/μL), median (IQR)			456 (303)	466 (299)	462 (301)
**Latest CD4 count (cells/μL), n (%)**					
		0-199	337 (9.29)	514 (8.17)	851 (8.58)
		200-499	1765 (48.65)	3020 (47.98)	4785 (48.23)
		≥500	1526 (42.06)	2760 (43.85)	4286 (43.2)
**Latest HIV load (copies/mL), n (%)**					
		0-49	3374 (93)	5900 (93.74)	9274 (93.47)
		50-999	115 (3.17)	170 (2.7)	285 (2.87)
		≥1000	139 (3.83)	224 (3.56)	363 (3.66)
Time since ART^a^ initiation (years), median (IQR)			5.35 (4.65)	5.35 (4.66)	4.65 (4.65)
**Time since ART initiation (years), n (%)**					
		0	139 (3.83)	237 (3.77)	376 (3.79)
		1-3	343 (9.45)	663 (10.53)	1006 (10.14)
		2-4	1131 (31.17)	2019 (32.08)	3150 (31.75)
		≥5	2015 (55.54)	3375 (53.62)	5390 (54.32)
**TDF^b^ use, n (%)**					
		No	1281 (35.31)	2537 (40.31)	3818 (38.48)
		Yes	2347 (64.69)	3757 (59.69)	6104 (61.52)
**3TC^c^ use, n (%)**					
		No	363 (10.01)	938 (14.9)	1301 (13.11)
		Yes	3265 (89.99)	5356 (85.1)	8621 (86.89)
**EFV^d^ use, n (%)**					
		No	1072 (29.55)	2202 (34.99)	3274 (33)
		Yes	2556 (70.45)	4092 (65.01)	6648 (67)
**AZT^e^ use, n (%)**					
		No	2804 (77.29)	4935 (78.41)	7739 (78)
		Yes	824 (22.71)	1359 (21.59)	2183 (22)
**LPV/r^f^ use, n (%)**					
		No	3311 (91.26)	5812 (92.34)	9123 (91.95)
		Yes	317 (8.74)	482 (7.66)	799 (8.05)
**Time since last vaccination (months), n (%)**					
		No vaccination	539 (14.86)	991 (15.75)	1530 (15.42)
		0-2	170 (4.69)	285 (4.53)	455 (4.59)
		3-5	96 (2.65)	141 (2.24)	237 (2.39)
		6-11	1734 (47.79)	2973 (47.24)	4707 (47.44)
		≥12	1089 (30.02)	1904 (30.25)	2993 (30.17)
**Vaccination status, n (%)**					
		No vaccination	539 (14.86)	991 (15.75)	1530 (15.42)
		Partial vaccination	101 (2.78)	156 (2.48)	257 (2.59)
		Full primary vaccination	545 (15.02)	975 (15.49)	1520 (15.32)
		Booster vaccination	2443 (67.34)	4172 (66.29)	6615 (66.67)

^a^ART: antiretroviral treatment.

^b^TDF: tenofovir.

^c^3TC: lamivudine.

^d^EFV: efavirenz.

^e^AZT: azidothymidine.

^f^LPV/r: lopinavir and ritonavir.

### SARS-CoV-2 Infection and Associated Factors Among People Living With HIV Who Had a COVID-19 Testing History in Zhejiang Province From December 2022 to February 2023

Among people living with HIV with a COVID-19 testing history, the SARS-CoV-2 infection rate was 66.97% (95% CI 65.81%-68.13%). The SARS-CoV-2 infection rate was significantly higher in the younger age group, those with single or divorced status, those with homosexual transmission routes, those with higher education levels, those with higher latest CD4 counts, those with lower latest HIV viral load, those not taking TDF, 3TC, and EFV, and those taking AZT, and those who had a long time since their last vaccination (Table 2). Multivariate logistic regression analysis revealed that age, education, transmission route, latest CD4 count, and latest HIV viral load were statistically associated with SARS-CoV-2 infection. Regarding vaccination, compared to those who had not received any vaccination, there was no statistically significant difference among those who received vaccination 4–6 months and 7-12 months since their last dose. However, there was a statistically significant association among those who had been vaccinated for 0-3 months (aOR 0.74, 95% CI 0.56-0.98) and more than 12 months (aOR 1.20, 95% CI 1.01-1.43; Table 2).

**Table 2:** SARS-CoV-2 infection and associated factors analysis among people living with HIV with a COVID-19 testing history in Zhejiang province from December 2022 to February 2023.

Variable			Infection					Multivariable analysis				
			People living with HIV, n (%)		*P* value			aOR^a^ (95% CI)			*P* value	
**Age categories (years)**						.001						
	15-29	754 (78.95)					Reference			Reference		
	30-39	1272 (76.12)					0.78 (0.64-0.96)			.02		
	40-49	825 (65.79)					0.51 (0.41-0.63)			.001		
	50-59	865 (61.92)					0.48 (0.38-0.59)			.001		
	≥60	499 (49.07)					0.31 (0.25-0.40)			.001		
**Sex**						.77						
	Male	3475 (67.05)					—^b^			—		
	Female	740 (66.61)					—			—		
**Education**						.001						
	College degree or above	112 (49.34)					Reference			Reference		
	Illiteracy	571 (53.62)					0.66 (0.48-0.91)			.01		
	Primary school	1434 (65.33)					0.68 (0.55-0.84)			.001		
	Middle school	982 (72.42)					0.82 (0.70-0.98)			.03		
	High school or technical secondary school	1116 (76.91)					0.94 (0.79-1.12)			.49		
**Marital status**						.001						
	Single or divorced	2511 (69.96)					Reference			Reference		
	Married or cohabiting	1696 (62.93)					1.23 (1.08-1.40)			.001		
	Unknown	8 (80)					2.33 (0.47-11.6)			.30		
**Household registration**						.19						
	Zhejiang province	2893 (66.44)					—			—		
	Other provinces	1322 (68.14)					—			—		
**Hypertension**						.14						
	No	364 (64.20)					—			—		
	Yes	3851 (67.24)					—			—		
**Diabetes**						.12						
	No	164 (62.60)					—			—		
	Yes	4051 (67.16)					—			—		
**Chronic obstructive pneumonia**						.11						
	No	16 (84.21)					—			—		
	Yes	4199 (66.92)					—			—		
**Cardiovascular diseases**												
	No	48 (76.19)		.12			—			—		
	Yes	4167 (66.88)					—			—		
**Chronic kidney disease**						.23						
	Yes	21 (77.78)					—			—		
	No	4194 (66.92)					—			—		
**Cancer**						.12						
	Yes	30 (78.95)					—			—		
	No	4185 (66.90)					—			—		
**Cerebrovascular disease**						.97						
	Yes	18 (66.67)					—			—		
	No	4197 (66.97)					—			—		
**Transmission**						.001						
	Heterosexual	2142 (61.15)					Reference			Reference		
	Homosexual	2033 (74.39)					1.30 (1.15-1.48)			.001		
	Other or unknown	40 (68.97)					1.30 (0.73-2.31)			.37		
**Latest CD4 count (cells/μL)**						.001						
	0-199	282 (54.86)					Reference			Reference		
	200-499	2034 (67.35)					1.35 (1.10-1.65)			.004		
	≥500	1899 (68.80)					1.22 (0.99-1.50)			.07		
**Latest HIV load (copies/mL)**						.001						
	0-49	3984 (67.53)					Reference			Reference		
	50-999	107 (62.94)					0.90 (0.65-1.26)			.55		
	≥1000	124 (55.36)					0.68 (0.51-0.92)			.01		
**Time since ART^c^ initiation (years)**						.001						
	0	156 (65.82)					Reference			Reference		
	1	407 (61.39)					0.85 (0.61-1.19)			.35		
	2-4	1325 (65.63)					1.08 (0.79-1.48)			.61		
	≥5	2327 (68.95)					1.27 (0.93-1.73)			.13		
**TDF^d^ use**						.001						
	No	1825 (71.94)					Reference			Reference		
	Yes	2390 (63.61)					0.99 (0.76-1.29)			.93		
**3TC^e^ use**												
	No	718 (76.55)		.001			Reference			Reference		
	Yes	3497 (65.29)					0.60 (0.45-0.79)			.001		
**EFV^f^ use**						.001						
	No	1535 (69.71)					Reference			Reference		
	Yes	2680 (65.49)					1.06 (0.91-1.23)			.45		
**AZT^g^ use**						.001						
	No	3256 (65.98)					Reference			Reference		
	Yes	959 (70.57)					1.23 (0.93-1.62)			.141		
**LPV/r^h^ use**						.32						
	No	3902 (67.14)					—			—		
	Yes	313 (64.94)					—			—		
**Time since last vaccination (months)**						.001						
	No vaccination	633 (63.87)					Reference			Reference		
	0-2	159 (55.79)					0.74 (0.56-0.98)			.04		
	3-5	81 (57.45)					0.80 (0.55-1.17)			.25		
	6-11	1990 (66.94)					1.07 (0.91-1.25)			.43		
	≥12	1352 (71.01)					1.20 (1.01-1.43)			.03		

^a^aOR: adjusted odds ratio.

^b^Not available.

^c^ART: antiretroviral treatment.

^d^TDF: tenofovir.

^e^3TC: lamivudine.

^f^EFV: efavirenz.

^g^AZT: azidothymidine.

^h^LPV/r: lopinavir and ritonavir.

### Association Between Vaccination and SARS-CoV-2 Infection Among Different Vaccination Statuses Across Different Times Since the Last Vaccination Among People Living With HIV With a COVID-19 Testing History in Zhejiang Province From December 2022 to February 2023

**Figure 2:**
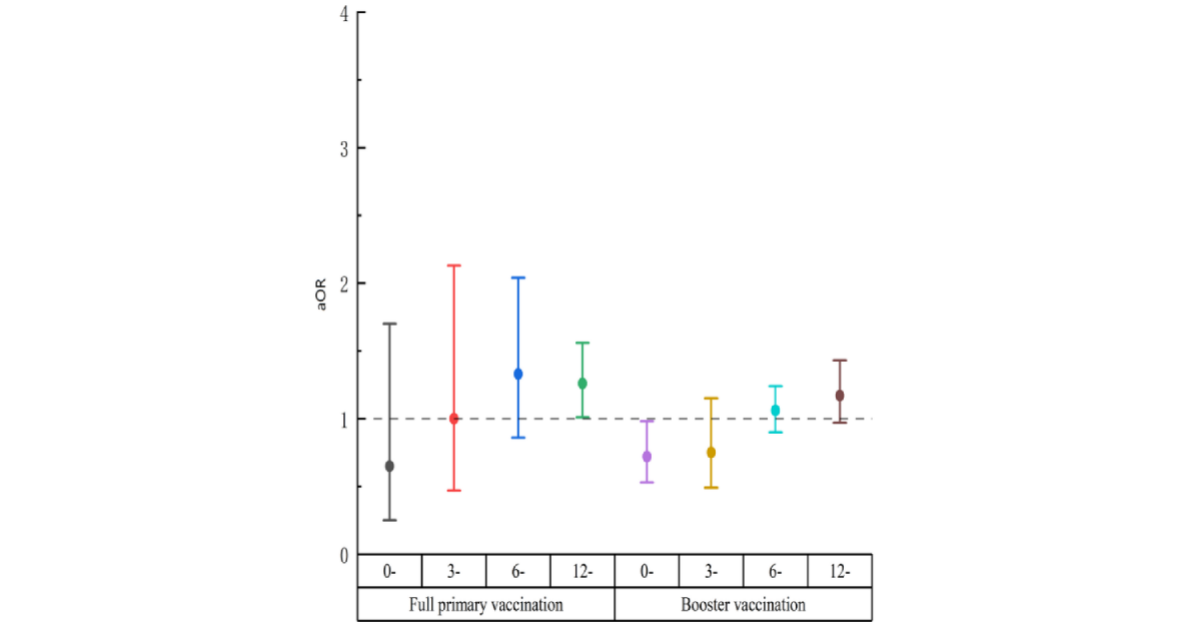
Association between vaccination and SARS-CoV-2 infection among different vaccination statuses across different times since the last vaccination among people living with HIV with a COVID-19 testing history in Zhejiang province from December 2022 to February 2023. aOR: adjusted odds ratio.

Among people living with HIV with a COVID-19 testing history in the full primary participants, there was no statistically significant association between SARS-CoV-2 infection and vaccination interval for those less than 3 months, 3-5 months, and 6-11 months. However, there was a statistically significant association between SARS-CoV-2 infection and vaccination for those vaccinated 12 months or more ago (aOR 1.26, 95% CI 1.01-1.56; Figure 2).

Regarding the booster vaccination among people living with HIV, there was a statistically significant association between SARS-CoV-2 infection and vaccination for those who were vaccinated <3 months (aOR 0.72, 95% CI:0.53–0.98). However, no statistical associations between SARS-CoV-2 infection and vaccination were observed for other conditions (Figure 2).

### COVID-19 Hospitalization and Associated Factors Among People Living With HIV Who Had Positive Tests for COVID-19 in Zhejiang Province From December 2022 to February 2023

Among the 4215 confirmed COVID-19 cases, 30 individuals (0.71%, 95% CI 0.46%-0.97%) required hospitalization. The highest hospitalized rates were observed among individuals aged ≥60 years, those with illiteracy, married individuals, individuals with hypertension, diabetes, chronic kidney disease, or cancer, individuals with the latest CD4 count below 200 cells/μL, an HIV load greater than 1000 copies/mL, and those who had not received any vaccination (Table 3).

In the multivariate analysis, several factors, including age, cancer, latest CD4 count, latest HIV viral load, and time since the last vaccination, remained significantly associated with COVID-19 hospitalization. Compared to people living with HIV aged <30 years, those aged >60 years showed an increased risk of hospital admission (aOR 7.44, 95% CI 1.23-45.17). Hospital admission was also associated with having cancer (aOR 12.19, 95% CI 2.98-49.93). Furthermore, compared to individuals who had not received any vaccination, people living with HIV who had received vaccination in the past 6-12 months (aOR 0.33, 95% CI 0.14-0.81) and those who had received vaccination more than 12 months ago (aOR 0.22, 95% CI 0.07-0.72) had a lower risk of hospitalization (Table 3).

**Figure 3:**
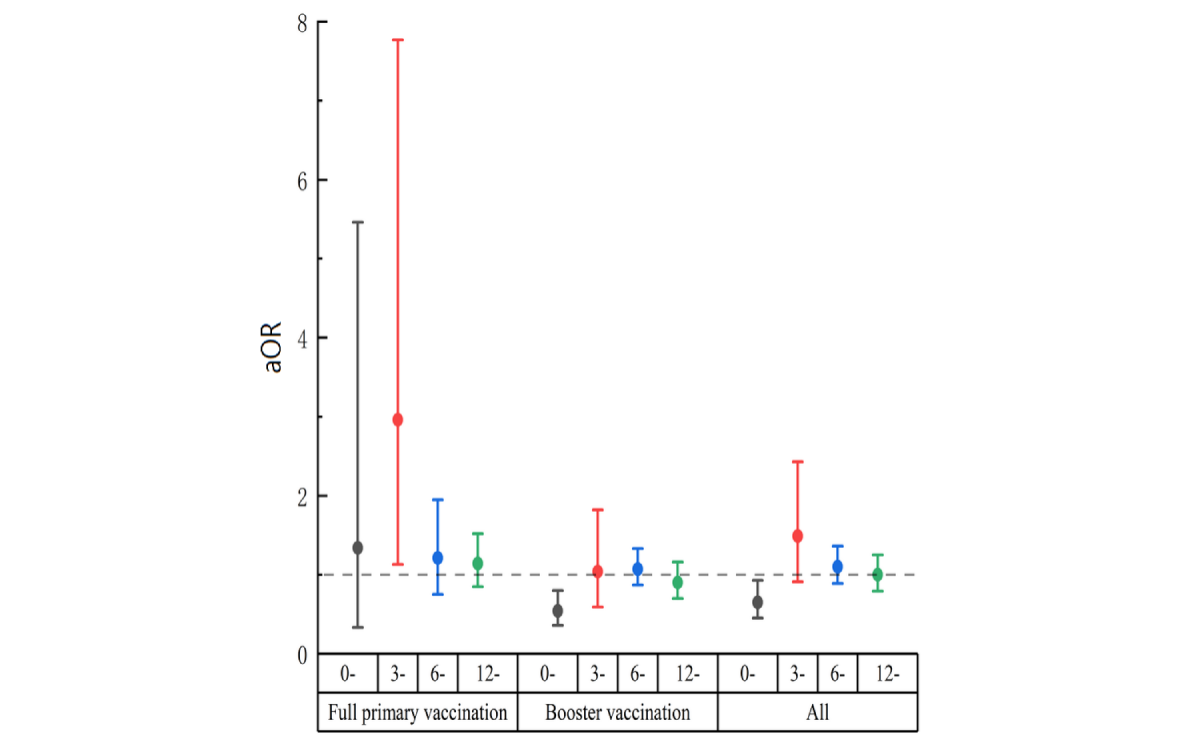
Association between vaccination and SARS-CoV-2 infection among group A in Zhejiang province from December 2022 to February 2023 (group A definition: those who presented with symptoms such as fever or cough were classified as having SARS-CoV-2 infection, while those who did not exhibit such symptoms were categorized as noninfected). aOR: adjusted odds ratio.

**Figure 4:**
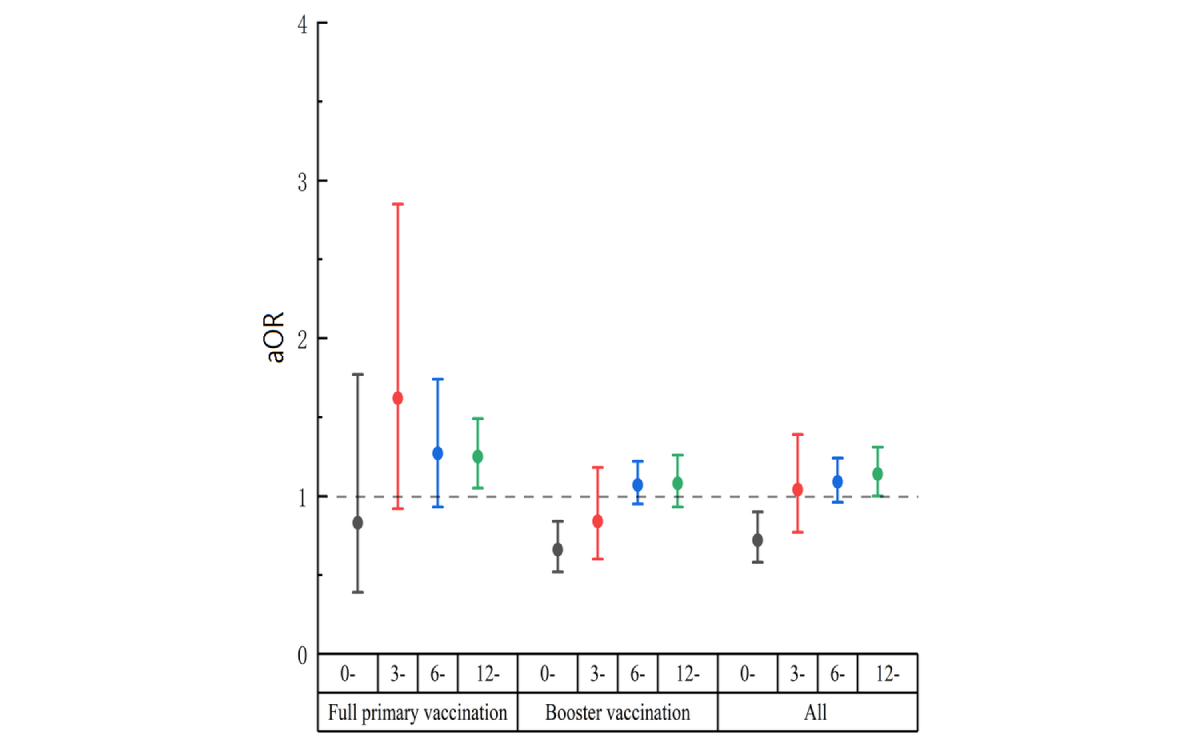
Association between vaccination and SARS-CoV-2 infection among group C in Zhejiang province from December 2022 to February 2023 (group C encompassed all study participants, including group A and group B. Group A definition: those who presented with symptoms such as fever or cough were classified as having SARS-CoV-2 infection, while those who did not exhibit such symptoms were categorized as noninfected. Group B comprised individuals who had undergone COVID-19 testing between December 7, 2022, and the investigation date. Infection status was determined based on the positive or negative results obtained from nucleic acid or antigen tests). aOR: adjusted odds ratio.

Sensitivity analyses were conducted among groups A and C to evaluate the robustness of our results. The SARS-CoV-2 infection rate was 67.03% (95% CI 65.50%-68.56%) and 66.99% (95% CI 66.07%-67.92%) in groups A and C, respectively. Moreover, as shown in Figures 3 and 4, there was a statistically significant association between SARS-CoV-2 infection and vaccination for those who were vaccinated <3 months in group A (aOR 0.65, 95% CI 0.45-0.93; Figure 3) and group C (aOR 0.72, 95% CI 0.58-0.90; Figure 4). Subgroup analysis showed that the association between vaccination and SARS-CoV-2 infection occurred at the booster vaccination while not in the full primary group (Figures 3 and 4).

**Table 3:** SARS-CoV-2 hospitalization and associated factors analysis among people living with HIV who had positive tests of COVID-19 in Zhejiang province from December 2022 to February 2023.

Variable		COVID-19 cases (n=4215), n (%)	Hospitalization			Multivariable analysis			
			People living with HIV, n (%)	*P* value		aOR^a^ (95% CI)		*P* value	
**Age categories (years)**					.001				
	15-29	754 (17.89)	2 (0.27)			Reference		Reference	
	30-39	1272 (30.18)	1 (0.08)			0.27 (0.02-3.10)		.29	
	40-49	825 (19.57)	2 (0.24)			0.45 (0.05-3.68)		.45	
	50-59	865 (20.52)	6 (0.69)			1.19 (0.19-7.49)		.85	
	≥60	499 (11.84)	19 (3.81)			7.44 (1.23-45.17)		.03	
**Sex**					.07				
	Male	3475 (82.44)	21 (0.6)			Reference		Reference	
	Female	740 (17.56)	9 (1.22)			1.39 (0.55-3.55)		.49	
**Education**					.001				
	College degree or above	112 (2.66)	5 (4.46)			Reference		Reference	
	Illiteracy	571 (13.55)	8 (1.4)			2.96 (0.43-20.3)		.27	
	Primary school	1434 (34.02)	9 (0.63)			0.83 (0.14-5.05)		.84	
	Middle school	982 (23.3)	6 (0.61)			1.04 (0.19-5.80)		.96	
	High school or technical secondary school	1116 (26.48)	2 (0.18)			1.72 (0.30-9.84)		.54	
**Marital status**					.001				
	Single or divorced	2519 (59.76)	7 (0.28)			Reference		Reference	
	Married or cohabiting	1696 (40.24)	23 (1.36)			0.45 (0.17-1.20)		.11	
**Household registration**					.08				
	Zhejiang province	2893 (68.64)	25 (0.86)			Reference		Reference	
	Other provinces	1322 (31.36)	5 (0.38)			1.17 (0.40-3.48)		.77	
**Hypertension**					.04				
	No	364 (8.64)	6 (1.65)			Reference		Reference	
	Yes	3851 (91.36)	24 (0.62)			0.71 (0.26-1.95)		.50	
**Diabetes**					.03				
	No	164 (3.89)	4 (2.44)			Reference		Reference	
	Yes	4051 (96.11)	26 (0.64)			1.10 (0.31-3.92)		.88	
**Chronic obstructive pneumonia**					>.99				
	No	16 (0.38)	0 (0)			—^b^		—	
	Yes	4199 (99.62)	30 (0.71)			—		—	
**Cardiovascular diseases**					>.99				
	No	48 (1.14)	0 (0)			—		—	
	Yes	4167 (98.86)	30 (0.72)			—		—	
**Chronic kidney disease**					.001				
	No	21 (0.5)	2 (9.52)			Reference		Reference	
	Yes	4194 (99.5)	28 (0.67)			1.10 (0.31-3.92)		.88	
**Cancer**					.001				
	No	30 (0.71)	4 (13.33)			Reference		Reference	
	Yes	4185 (99.29)	26 (0.62)			12.19 (2.98-49.93)		.001	
**Cerebrovascular disease**					>.99				
	No	18 (0.43)	0 (0)			—		—	
	Yes	4197 (99.57)	30 (0.71)			—		—	
**Transmission route**					.001				
	Heterosexual	2142 (50.82)	25 (1.17)			Reference		Reference	
	Homosexual	2033 (48.23)	4 (0.2)			0.57 (0.17-1.98)		.38	
	Other or unknown	40 (0.95)	1 (2.5)			4.26 (0.47-38.32)		.20	
**Latest CD4 count** (**cells/μL)**					.001				
	0-199	282 (6.69)	10 (3.55)			Reference		Reference	
	200-499	2034 (48.26)	8 (0.39)			0.13 (0.04-0.38)		.001	
	≥500	1899 (45.05)	12 (0.63)			0.39 (0.14-1.06)		.07	
**Latest HIV load** (**copies/mL)**					.02				
	0-49	3984 (94.52)	25 (0.63)			Reference		Reference	
	50-999	107 (2.54)	2 (1.87)			1.42 (0.23-8.87)		.71	
	≥1000	124 (2.94)	3 (2.42)			4.21 (1.01-17.60)		.049	
**Time since ART^c^ initiation (cells/μL)**					.88				
	0	156 (3.7)	1 (0.64)			—		—	
	1	407 (9.66)	4 (0.98)			—		—	
	2-4	1325 (31.44)	8 (0.6)			—		—	
	≥5	2327 (55.21)	17 (0.73)			—		—	
**TDF^d^ use**					.71				
	Yes	1825 (43.3)	14 (0.77)			—		—	
	No	2390 (56.7)	16 (0.67)			—		—	
**3TC^e^ use**					.96				
	Yes	718 (17.03)	5 (0.7)			—		—	
	No	3497 (82.97)	25 (0.71)			—		—	
**EFV^f^ use**					.43				
	Yes	1535 (36.42)	13 (0.85)			—		—	
	No	2680 (63.58)	17 (0.63)			—		—	
**AZT ^g^ use**					.42				
	Yes	3256 (77.25)	25 (0.77)			—		—	
	No	959 (22.75)	5 (0.52)			—		—	
**LPV/r^h^ use**					.28				
	Yes	3902 (92.57)	26 (0.67)			—		—	
	No	313 (7.43)	4 (1.28)			—		—	
**Time since last vaccination** (**months)**					.001				
	No vaccination	633 (15.02)	13 (2.05)			Reference		Reference	
	0-5	240 (5.69)	1 (0.42)			0.21 (0.03-1.73)		.15	
	6-11	1990 (47.21)	12 (0.6)			0.33 (0.14-0.81)		.02	
	≥12	1352 (32.08)	4 (0.3)			0.22 (0.07-0.72)		.01	

^a^aOR: adjusted odds ratio.

^b^Not available.

^c^ART: antiretroviral treatment.

^d^TDF: tenofovir.

^e^3TC: lamivudine.

^f^EFV: efavirenz.

^g^AZT: azidothymidine.

^h^LPV/r: lopinavir and ritonavir.

## Discussion

### Overview

The public health impact of the COVID-19 pandemic in the community continues. Previous studies have investigated the SARS-CoV-2 infection and hospitalization and their associated risk factors among people living with HIV [6,19,20]. However, most of these studies were conducted during the early stages of the pandemic or when the Delta variant was predominant. Furthermore, due to the limited number of infections resulting from implementing the Dynamic Zero policy, no studies regarding the Omicron variant have been reported in China. This study aimed to investigate SARS-CoV-2 infection and hospitalization among people living with HIV during the Omicron variant epidemic in China. The infection rate was 66.97% (4215/6294) among people living with HIV with a COVID-19 testing history, whereas the hospitalization rate was 0.71% (30/4215). Moreover, we found that general risk factors such as higher age, vaccination status, and HIV-related parameters such as latest CD4 count and HIV load were associated with SARS-CoV-2 infection and hospitalization, and these results will provide scientific information for future control and prevention of COVID-19 among people living with HIV.

The SARS-CoV-2 infection rate was 66.97% (4215/6294) in this study, which was lower than the rate reported by Fu et al [11] (82.4%), the rate estimated by modeling calculation conducted by Leung et al (92.3%) [14], and Bai et al (87.54%) [15], and higher than the rate investigated among children conducted by Su et al [12] (40.9%) in the general population after adjustment of the Zero-COVID policy. During the early pandemic, studies mostly demonstrated a lower or similar incidence of SARS-CoV-2 infection among people living with HIV when compared to those without HIV in the general population [4]. The lower infection rate among people living with HIV could be due to the following 2 reasons: first, many studies have indicated that COVID-19 has a great impact on people living with HIV, and thus they usually have greater social distancing [4,21]. Second, some researchers have argued that antiretroviral medication, specifically nucleoside or nucleotide reverse transcriptase inhibitors, may have contributed to a protective effect [8,22]. However, the lower rate can be attributed to other factors such as sample size, confounding biases, and geographical, social, and health care disparities, which should be concluded with caution and deserve further study.

We found that age, education, marital status, transmission route, latest CD4 cell count, and HIV viral load were statistically associated with SARS-CoV-2 infection. Younger adults had a higher risk of SARS-CoV-2 infection, potentially due to a higher force of infection in working age groups; this result was consistent with a study conducted among the general population in Shanghai [17] and people living with HIV in Lebanon [8]. In this study, being married or cohabiting was independently associated with infection, which could be related to intrafamily transmission. Furthermore, consistent with the study conducted by Kim and Jeong [23], we found that the higher the education level, the higher the SARS-CoV-2 infection rate. Interestingly, people living with HIV with higher latest CD4 counts had a higher risk of infection, while those with higher HIV loads had a lower risk. This phenomenon may also contribute to illustrating the lower infection rate in people living with HIV in our study compared to studies in the general population. Nonetheless, the reasons for this phenomenon require further investigation. In addition, people living with HIV who contracted HIV from homosexual transmission had a higher risk of SARS-CoV-2 infection than those who contracted HIV from heterosexual transmission. This higher risk may be attributed to higher mobility and multiple sexual partners among men who have sex with men, which may increase their risk of infection [24-26].

According to a World Health Organization report, people living with HIV have a significantly higher risk of experiencing a severe or fatal COVID-19 infection [27]. Consistent with previous studies, we identified several factors associated with an increased risk of hospitalization among people living with HIV. These factors include older age, lower latest CD4 count, higher latest HIV viral load, and the presence of cancer [3,6,7,19,28]. Therefore, people living with HIV with these risk factors should be given priority care and protection.

In this study, we found 3TC had a protective effect against SARS-CoV-2 infection, and this protective effect was not shown in other commonly used drugs (TDF, EFV, AZT, and LPV/r). Previous studies have demonstrated that there was a molecular basis for the inhibition of the SARS-CoV-2 RNA-dependent RNA polymerase by these nucleotide analogs [29,30]. However, the effectiveness of antiretrovirals in preventing COVID-19 infection or severe illness among people living with HIV has yielded mixed results in different studies and trials [8,22,31]. Therefore, it is currently impossible to definitively conclude whether antiretrovirals can protect people from contracting COVID-19 or experiencing severe illness from the virus.

Data on the effectiveness of COVID-19 vaccines among people living with HIV in real-world studies remain limited, as they have not been adequately represented in initial vaccine effectiveness trials. In our study, we found that vaccination was associated with reduced SARS-CoV-2 infection within 3 months of the last vaccination. Our subgroup analyses further demonstrated that the protective effect only occurred in the booster vaccination participants. Our findings were consistent with the vaccine effectiveness of COVID-19 evaluated among the general population in China [11]. Furthermore, the significant effect of vaccination in reducing hospitalization in our study aligns with observations made in the general population and among people living with HIV in other studies [17,32]. Notably, the duration of protection against hospitalization varied and may be attributed to the small sample size of hospitalized cases. Since no studies specifically focused on vaccination efficacy against SARS-CoV-2 Omicron infection and hospitalization among the population of people living with HIV in the country, our study provided a scientific basis for the vaccination program among people living with HIV. Consequently, we highly recommend expanding vaccination coverage among people living with HIV when the epidemic reemerges since 15% (1530/9922) of people living with HIV in our study remained unvaccinated.

### Limitations

This study had several limitations. Firstly, a causal relationship could not be established between the investigated factors and the infection or hospitalization of SARS-CoV-2 due to the cross-sectional nature of our study. Secondly, although we used random sampling at the beginning of participant selection, the inability to collect data from refusal would lead to selection bias. Our findings should be extrapolated with caution. Thirdly, diagnoses of conditions such as SARS-CoV-2 infection, hospitalization, and other chronic diseases such as diabetes, hypertension, and cardiovascular disease rely on self-reporting, which can be influenced by social desirability and reporting biases. Fourthly, other potential risk factors for infection, such as individual behaviors and close contact infections, which may impact infection, were not investigated. Finally, the number of hospitalizations was too small to conduct subgroup analyses and further estimations of the effectiveness of different vaccination strategies against hospitalization.

### Conclusions

We conducted the first investigation of HIV/SARS-CoV-2 coinfection and hospitalization following the ease of the control policy in China. Our findings revealed a high prevalence of COVID-19 but a low hospitalization rate. Besides general characteristics such as age and vaccination status, we also found that people living with HIV–specific factors such as CD4 cell count and HIV viral load were associated with SARS-CoV-2 infection and hospitalization. These findings will add to the growing literature on SARS-CoV-2 and HIV coinfection and contribute to optimizing COVID-19 preventive strategies among people living with HIV. A booster vaccination campaign for booster doses should be considered among people living with HIV in confronting possible COVID-19 epidemic emergencies in the near future.

## Abbreviations

3TC: lamivudine
aOR: adjusted odds ratio
ART: antiretroviral treatment
AZT: azidothymidine
CDC: Center for Disease Control and Prevention
CHO cell: Chinese hamster ovary cell
EFV: efavirenz
LPV/r: lopinavir and ritonavir
TDF: tenofovir

## Data Availability

The data analyzed in our study are available from the corresponding author on reasonable request.
